# Construction of a conceptual model and preliminary content development for patient-reported outcomes measurement after total hip arthroplasty: from a Chinese perspective

**DOI:** 10.3389/fmed.2026.1753638

**Published:** 2026-05-25

**Authors:** Liang Li, Weiwei Sun, Wei Zhang, Yongfeng Chen, Jianhui Zhai, Dawei Zhang, Jianbing Ma, Yuhai Zhang, Chao Xu, Lei Shang

**Affiliations:** 1 Department of Health Statistics, School of Public Health, The Fourth Military Medical University, Xi’an, Shaanxi, China; 2 Ministry of Education Key Lab of Hazard Assessment and Control in Special Operational Environment, Xi’an, Shaanxi, China; 3 The Jingxi Medical District of the PLA General Hospital, Beijing, China; 4 Department of Orthopedics, Xijing Hospital, Air Force Medical University, Xi’an, China; 5 Department of Hip Joint Surgery, Honghui Hospital, Xi’an Jiaotong University, Xi’an, China; 6 Department of Knee Joint Surgery, Honghui Hospital, Xi’an Jiaotong University, Xi’an, China

**Keywords:** arthroplasty, conceptual model, Delphi method, hip, patient reported outcome measures

## Abstract

**Aim:**

This study aims to develop a conceptual model and preliminary content for patients who have undergone total hip arthroplasty (THA) in Mainland China.

**Methods:**

Guided by the biopsychosocial medical model, this study was designed in three phases: (1) conduct a targeted literature review and construct an item pool; (2) collect qualitative data from experts and patients to develop a preliminary Chinese version of the patient-reported outcome measurement (PROM) for THA; and (3) have experts review the Chinese THA PROM (CTP) using the Delphi method, followed by cognitive debriefing interviews with patients.

**Results:**

A total of 50 patients and 17 experts participated in this study. The constructed conceptual model focused on eight key dimensions: function, pain, symptoms, quality of life, cognitive and social support, psychology, expectations, and satisfaction. A total of 99 items were developed for the corresponding CTP.

**Conclusion:**

With extensive participation from patients and multidisciplinary experts, the conceptual model and preliminary content of the CTP were developed. By integrating patient perspectives and clinical practice, the CTP comprehensively addresses disease experiences and clinical focuses. Following further refinement through psychometric testing, the CTP is expected to become a standardized and comprehensive measurement tool for research on Chinese patients who have undergone THA.

## 1 Introduction

Total hip arthroplasty (THA) is a surgical procedure in which an artificial hip joint prosthesis is used to replace a diseased hip joint. The main indications for THA include advanced hip arthritis, femoral head necrosis, femoral neck fractures in elderly patients, poor healing after femoral neck fracture surgery, and congenital hip dysplasia (1). THA can significantly relieve hip pain and enhance joint mobility. As reported by the Chinese guidelines for the diagnosis and treatment of osteoarthritis (2021 edition), the ten-year survival rate of the prosthesis after THA has reached 95.6%, while the twenty-year survival rate remains at 85% (2). THA has become one of the most successful surgeries in orthopedic medicine (3). Since THA is an elective procedure, postoperative satisfaction cannot be assessed solely based on objective clinical indicators. It is important to apply Patient-Reported Outcome Measurements (PROMs) to assess the postoperative functional experience and subjective perception of patients (4).

Compared to traditional clinical evaluation methods, PROMs (5) can help reduce potential subjective biases from doctors during the evaluation process, resulting in more objective and accurate results. PROMs also capture the issues and need that matter most to patients, making medical services more considerate and personalized. Furthermore, through PROMs, effective follow-up can be conducted even if patients are unable to visit medical institutions in person. Medical institutions can adjust treatment plans and intervention measures in a timely manner based on the PROMs data provided by patients, thereby better meeting their needs.

At present, Chinese-translated versions of PROMs originally developed in Western countries are widely used to evaluate outcomes in patients undergoing THA in China. However, substantial differences exist between China and Western countries with respect to cultural background, lifestyle, health beliefs, and socioeconomic context. The direct application of these instruments without adequate cultural adaptation may compromise the accuracy and validity of postoperative assessments. Therefore, there is a pressing need to develop a culturally specific PROM tailored to Chinese patients undergoing THA (6).

The conceptual model defines the meanings and scopes of the measurement dimensions of the scale, and a scientifically sound conceptual model is essential for developing effective and reliable measurement tools (7). However, due to the lack of a widely accepted conceptual model for the PROMs of THA patients, the PROMs instruments used in clinical practice are inconsistently structured, leading to non-comparable assessment results (8). This study aims to construct a conceptual model and develop preliminary content for PROM for Chinese patients undergoing THA.

## 2 Materials and method

### 2.1 Design

This study was divided into three stages: literature review, conceptual elicitation, and content validity assessment. All patients enrolled in this study either planned to undergo THA within 6 months or had undergone THA for at least 1 year. The study adhered to the principles of the Declaration of Helsinki and obtained ethical approval. All participants provided written informed consent.

#### 2.1.1 Sample size considerations

Qualitative sampling was purposive and aimed at maximum variation across age, sex, diagnosis, and postoperative time. For patient concept elicitation, we conducted one focus group (*n* = 10) followed by individual interviews (*n* = 20), and recruitment continued until thematic saturation was achieved (i.e., no new concepts relevant to item generation emerged) (9). For cognitive debriefing, we recruited 20 patients to ensure adequate representation of older adults and participants with lower educational attainment.

For the Delphi expert consultation phase of this study, the sample size was determined with reference to the 2019 consensus-based standards for the selection of health measurement instruments (COSMIN) Study Design Checklist for Content Validity of PROMs (10). Per the checklist recommendations, a robust content validity evaluation should recruit experts from all relevant disciplines, with a minimum of 4 experts required for purely qualitative studies and at least 30 experts for purely quantitative studies. However, the Delphi expert consultation is a mixed-methods approach integrating both qualitative and quantitative research characteristics; thus, the aforementioned threshold criteria for single-type research designs are not applicable to this methodology. Systematic literature review confirmed that there is currently no unified mandatory standard for the number of experts selected for Delphi surveys internationally, and the appropriate sample size is usually determined according to specific research objectives. Combined with the core research goals, multidisciplinary coverage requirements, and implementation feasibility of this study, a sample size of 15–20 experts was pre-specified as appropriate. Accordingly, a total of 20 multidisciplinary experts were invited to participate in the Delphi expert consultation. Valid responses were collected from 17 experts in the first round and 15 experts in the second round. This panel size fully complies with internationally recognized standards for content validity validation of PROMs via the Delphi method. The expert attrition between rounds and its potential impact on the study results have been elaborated in detail in the Results and Limitations sections of this manuscript.

##### 2.1.1.1 Phase I: scoping review

At present, a wide array of THA-related PROMs have been developed, covering multiple core domains including function, pain, and psychological status. However, the existing literature lacks a systematic synthesis of their measurement properties (including reliability, validity, responsiveness, etc.), clinical application scenarios, and dimensional structure. The core objective of this study was to construct a conceptual framework for the development of localized THA-specific PROMs, rather than to evaluate the efficacy of specific interventions. Accordingly, a scoping review methodology was adopted.

A comprehensive literature search was conducted using English Medical Subject Headings (MeSH) terms and corresponding free-text terms, including “patient outcome assessment,” “structural validity,” “factor analysis,” “Cronbach’s alpha,” “cross-cultural,” “construct validity,” “minimal clinically important difference,” “limits of agreement,” “hip,” and “arthroplasty,” across four English databases: PubMed, Embase, Web of Science, and Scopus. Corresponding Chinese keywords were used to perform the search in three Chinese academic databases: China National Knowledge Infrastructure (CNKI), Wanfang Data Knowledge Service Platform, and VIP Chinese Journal Service Platform. The search time frame was set from the inception of each database to December 2024.

#### 2.1.2 Inclusion criteria

(1) Study participants were patients aged > 18 years; (2) patients were scheduled to undergo THA within 6 months or were at least 1 year post-THA (the postoperative time frame was defined to focus on the assessment of long-term/stable outcomes via PROMs and to avoid interference from short-term perioperative outcomes); (3) studies focused on THA-related PROMs covering domains including function, psychological status, quality of life, and pain, and reported the measurement properties of the investigated PROMs; (4) studies employed quantitative designs (descriptive, explanatory-predictive, correlational, etc.), qualitative designs (content analysis, theory-guided, etc.), or mixed-methods designs; (5) published peer-reviewed journal articles or reviews, with no language restrictions.

#### 2.1.3 Exclusion criteria

(1) studies focusing on patients with comorbidity-related conditions; (2) studies that assessed functional outcomes exclusively; (3) studies involving only body composition measurement; (4) conference abstracts, duplicate publications, articles with unavailable full text, and studies with incomplete information.

After removing duplicate literature, two independent reviewers (LL and WS) screened the titles and abstracts of all retrieved studies to identify those meeting the pre-specified criteria. The full texts of the candidate studies were subsequently reviewed to confirm the final included studies. To minimize the omission of relevant literature, a manual backward citation search was performed on the reference lists of all included studies. Any discrepancies between the two reviewers were resolved through discussion. The domains and items of the PROMs that met the inclusion criteria were extracted to establish a conceptual model library. The findings from this study phase provided the foundational basis for the development of the semi-structured interview guide.

##### 2.1.3.1 Phase II: concept elicitation among patients who have undergone THA and experts in related fields

We developed a semi-structured interview guide for one-on-one interviews with experts in THA-related fields. Each interview lasted approximately 60 min. During these in-depth sessions, the experts reviewed the Conceptual Model Pool (CMP), provided feedback on wording, organization, and content, discussed the impacts of life after THA, and considered other elements of the PROM, such as questionnaire length and ideal response options. The interviews were audio-recorded and transcribed verbatim. Two members of the research team (LL and WS) conducted the interviews, and the transcripts were coded and analyzed. Additionally, a semi-structured interview guide was developed for subsequent focus group meetings and individual interviews with THA patients.

On December 25, 2024, a face-to-face focus group interview with THA patients was conducted at Xi’an Honghui Hospital for approximately 90 min, followed by individual interviews. During the interviews, participants discussed the PROMs dimensions and item pool, as well as the impact of their conditions on daily life. The discussion process was recorded and transcribed verbatim. Two members of the research team (LL and CX) coded and analyzed the transcripts, focusing on identifying and elaborating on insights from the interviews. Additionally, the preferences of patients for various elements of the PROMs, such as the recall period and the ideal way and environment for completing the questionnaire, were also assessed.

#### 2.1.4 Qualitative data analysis

We conducted a thematic analysis using a hybrid inductive–deductive approach. A preliminary coding framework was informed by the biopsychosocial model and refined iteratively as new concepts emerged. Two researchers independently coded the transcripts, compared coding outputs, and reconciled discrepancies through discussion; unresolved disagreements were adjudicated by a senior member of the research team. The approach of reaching consensus to resolve disagreements complies with the requirements of the COSMIN Guidelines for qualitative analysis. Themes were generated by grouping codes into higher-order concepts and mapping them to candidate measurement dimensions and items.

The opinions and suggestions gathered from these interviews were used to develop the preliminary CTP, along with the item response options and instructions. All content was drafted in Chinese.

##### 2.1.4.1 Phase III: evaluation of the content validity of the PROM

This phase included two rounds of the Delphi method, followed by a formal cognitive debriefing interview. We adhered to the recommendations outlined by consensus-based recommendations for conducting and reporting Delphi studies (CREDES) (11).

### 2.2 Inclusion criteria

The experts who participated in this Delphi consultation were from multiple fields, including joint surgery, orthopedic nursing, rehabilitation medicine, medical statistics, sports medicine, orthopedic medical device research and development, and medical psychology. The experts were required to meet the following conditions: (1) having rich practical or research experience in fields related to THA, with at least 15 years of work experience; (2) holding a bachelor’s degree or higher; (3) having an intermediate or higher professional title; (4) having the enthusiasm to participate in this study; (5) abiding by the principle of informed consent.

### 2.3 Data collection

The principal investigators (LL, CX, and WS) contacted the experts via email or WeChat to distribute the expert consultation questionnaires (Table 1) and reminded them to provide feedback within 2 weeks. The researchers followed up with any experts who had not replied within that time frame to inquire about their progress.

**TABLE 1:** Content of expert consultation questionnaire.

Section	Content
Part 1: Investigation Introduction	(1) Research Overview: Background, purpose, and significance of the research;(2) Development Process: Steps involved in constructing dimensions and item pools;(3) Expert Consultation: Objectives of expert consultation, composition of the questionnaire, anticipated return time, and researchers’ contact information.
Part 2: Expert Information and Self-evaluation	(1) Basic information about the experts, including age, gender, work unit, years of experience, highest educational attainment, professional title, and main research field. (2) Familiarity with the consultation content and the basis for their judgments.
Part 3: Experts’ Evaluation and Suggestions on Dimensions and Items	(1) The first draft of dimensions and items; (2) Experts use a 5-point Likert scale to evaluate the rationality of dimensions and items: Very Important (5 points), Important (4 points), Moderate (3 points), Less Important (2 points), Unimportant (1 point); (3) Revision suggestions: Experts are invited to provide feedback on any dimensions and items, including additions, combinations, deletions, or modifications.

After the first round of questionnaires was collected, the researchers (LL and WS) analyzed the experts’ opinions and organized discussions among the research team. Items were considered for deletion or merging if the mean importance score was < 4.0 and/or the coefficient of variation (CV) was > 0.25, which are commonly used Delphi cut-offs to indicate insufficient perceived importance and poor consensus (12). Additionally, items were added or modified according to the feedback of the experts. Using the results from the first round of consultation, the second round of questionnaires was designed. The findings from the first round were included in the second round of questionnaires to help experts understand the rationale behind the modifications. The same procedures were followed for distributing and collecting the questionnaires. The consultation concluded when the experts reached consensus. In total, two rounds of expert consultations were conducted, with a 1-month interval between them.

Subsequently, a series of formal cognitive debriefing interviews were conducted with THA patients recruited from Xi’an Honghui Hospital. Two members of the research team (LL and JZ) carried out face-to-face interviews with each patient. These interviews followed a specially designed semi-structured interview guide aimed at assessing the relevance, comprehensibility, and comprehensiveness of the Chinese PROMs for THA patients. Participants utilized the concept testing protocol through the think-aloud method, a standardized cognitive debriefing approach that minimizes interviewer bias and provides valuable insights into participants’ understanding (13). The interview process was recorded and transcribed verbatim. LL and CX coded the transcripts and identified sections of the CTP that required revision.

### 2.4 Data analysis

All statistical analyses were conducted using SPSS version 24.0. The significance level was set at 0.05. The Kolmogorov–Smirnov test was employed to evaluate the normality of the data distribution, and it was determined that all data were normally distributed. The characteristics of the experts were presented as means and standard deviations, or frequencies and percentages. In the third stage, expert authority was assessed using the coefficient of expert authority (Cr), calculated as Cr = (Ca + Cs)/2 (14, 15). Here, Ca represents the coefficient of expert judgment awareness (i.e., the strength of the basis for judgment), which was derived from the self-rated reliance of the experts on practical experience, theoretical analysis, references, and intuition (Table 2). Cs represents the coefficient of expert judgment skill (i.e., familiarity with the consultation content), derived from the self-rated level of familiarity of the experts (Table 3). The centralization of the experts’ opinions was evaluated using the average importance score and the coefficient of variation (CV). The degree of coordination among experts was quantified using Kendall’s coefficient of concordance (W) (16). Specifically, for Ca, we applied a commonly used weighting scheme (practical experience = 0.5, theoretical analysis = 0.3, references = 0.1, intuition = 0.1), and each basis was scored as high/medium/low (1/0.5/0); Ca was calculated as the weighted sum. For Cs, familiarity was scored on a five-level scale (very familiar = 1.0, more familiar = 0.75, generally familiar = 0.5, less familiar = 0.25, unfamiliar = 0.0), and Cs was the assigned score.

**TABLE 2:** Self-evaluation by experts of the basis for their judgment.

Indicators	First round (*n* = 17)			Second round (*n* = 15)		
	High	Medium	Low	High	Medium	Low
Practical experience	10	6	1	10	4	1
theoretical analysis	10	7	0	9	6	0
references	8	6	3	7	5	3
intuition	2	5	11	2	4	9
Ca value	0.91 ± 0.07			0.92 ± 0.07		

**TABLE 3:** Self-evaluation by experts of the basis for their judgment.

Familiarity level	First round (*n* = 17)	Second round (*n* = 15)
Very familiar	10	10
More familiar	7	5
Generally familiar	0	0
Less familiar	0	0
Unfamiliar	0	0
Cs value	0.92 ± 0.10	0.93 ± 0.10

## 3 Results

**FIGURE 1:**
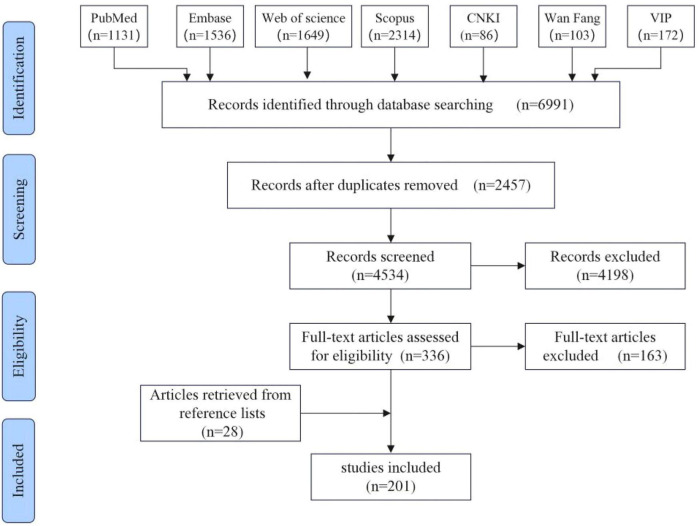
PRISMA flow diagram of the literature search and study selection process.

**FIGURE 2:**
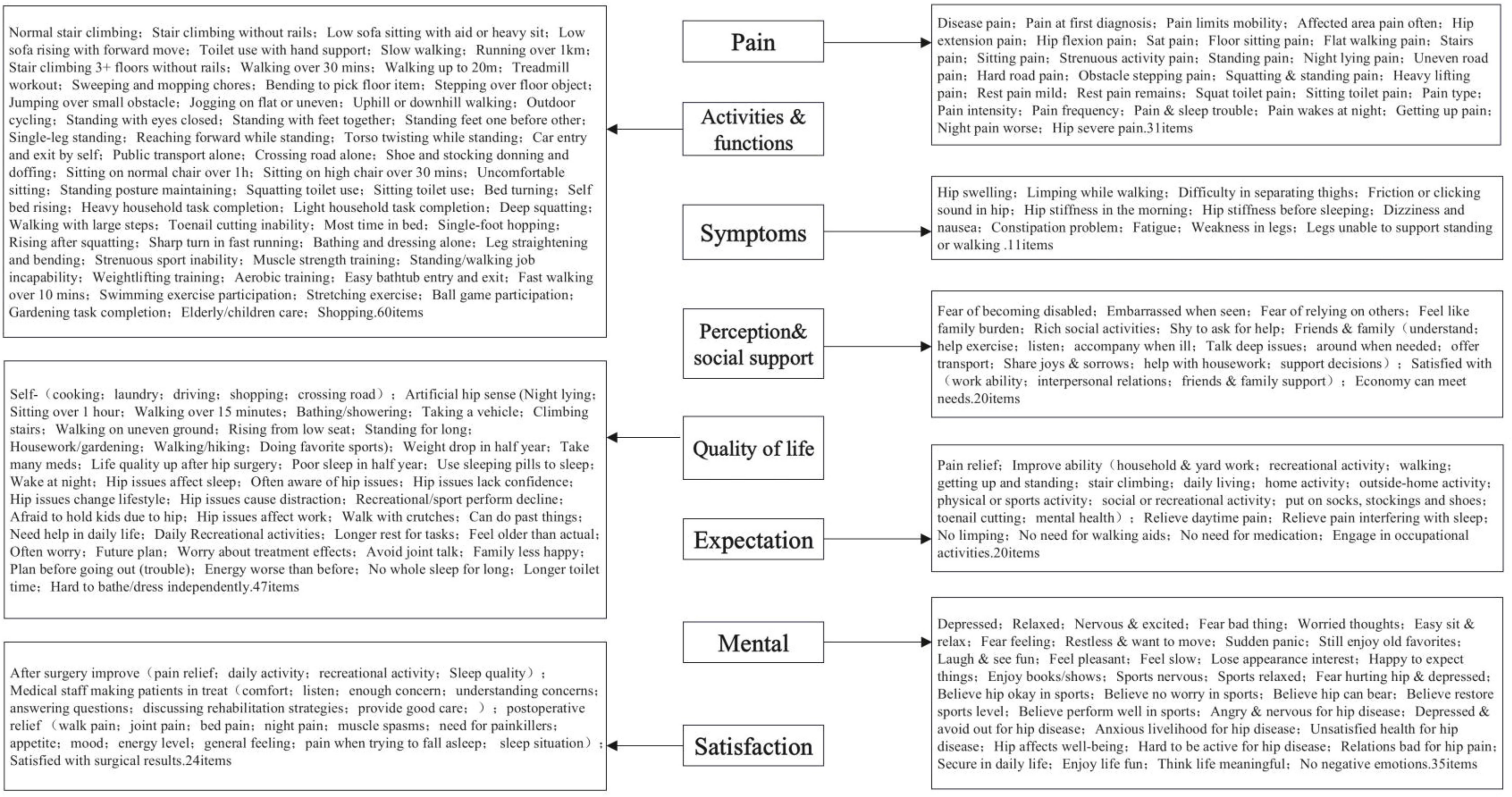
A conceptual model pool (CMP) for Chinese THA PROM(CTP).

Different participant groups took part in this study. Detailed professional and demographic information is presented in Tables 4, 5. Ultimately, 201 articles were selected from a total of 6,991 references (Figure 1). The research team identified and analyzed 48 PROMs (17–63). Based on the data extracted from the literature review, the team developed a list of eight dimensions and 248 items for the Conceptual Model Pool (CMP) (Figure 2).

**TABLE 4:** Basic information of experts in each stage.

Participant classification	Concept elicitation interviews (*N* = 9)	Delphi-round 1 (*N* = 17)	Delphi-round 2 (*N* = 15)
Age (Mean ± SD, years)	47.56 ± 2.24	43.00 ± 6.09	43.27 ± 6.46
Sex, n (%)			
Male	7 (77.78%)	13 (76.47%)	11 (73.33%)
Female	2 (22.22%)	4 (23.63%)	4 (26.67%)
Working experience (Mean ± SD, years)	23.56 ± 2.35	18.41 ± 6.97	19.00 ± 7.15
Research area, n (%)			
Joint surgery	6 (66.67%)	9 (52.94%)	7 (46.67%)
Orthopedic nursing	1 (11.11%)	2 (11.76%)	2 (13.33%)
Rehabilitation medicine	1 (11.11%)	3 (17.65%)	3 (20.00%)
Statistics	0 (0.00%)	1 (5.88%)	1 (6.67%)
Medical device development	0 (0.00%)	1 (5.88%)	1 (6.67%)
Medical psychology	1 (11.11%)	1 (5.88%)	1 (6.67%)

**TABLE 5:** Basic information of patients in each stage.

	Concept elicitation		Cognitive debriefing
Participant classification	Patient focus group (*N* = 10)	Individual interviews (*N* = 20)	Patient interviews (*N* = 20)
Age (Mean ± SD, years)	56.70 ± 10.13	64.60 ± 5.78	65.30 ± 7.24
Sex, n (%)			
Male	3 (30.00%)	6 (30.00%)	6 (30.00%)
Female	7 (70.00%)	14 (70.00%)	14 (70.00%)
Occupation			
Manual labor	2 (20.00%)	8 (40.00%)	7 (35.00%)
Non-manual labor	8 (80.00%)	12 (60.00%)	13 (65.00%)
Employment status			
In work	5 (50.00%)	5 (25.00%)	4 (20.00%)
Be unemployed	1 (10.00%)	8 (40.00%)	5 (25.00%)
Retired	4 (40.00%)	7 (35.00%)	11 (55.00%)
Living situation			
Living alone	0 (0.00%)	1 (5.00%)	1 (5.00%)
With spouse	7 (70.00%)	8 (40.00%)	10 (50.00%)
With children	0 (0.00%)	3 (15.00%)	5 (25.00%)
Children and spouse	3 (30.00%)	6 (30.00%)	3 (15.00%)
With care worker	0 (0.00%)	2 (10.00%)	1 (5.00%)
Education			
Primary school or below	0 (0.00%)	4 (20.00%)	1 (5.00%)
Middle school	2 (20.00%)	6 (30.00%)	8 (40.00%)
High school or above	2 (20.00%)	3 (15.00%)	6 (30.00%)
Junior college	4 (40.00%)	5 (25.00%)	3 (15.00%)
University	2 (20.00%)	2 (10.00%)	2 (10.00%)

Twelve experts participated in the concept elicitation interviews, during which they reviewed the Conceptual Model Pool and provided suggestions for the item settings. As a result, a total of 46 items with similar semantics, as well as those not aligned with the Chinese lifestyle, were deleted (Supplementary Appendix 1).

Ten THA patients participated in the concept elicitation focus group and confirmed the revised list of dimensions and items. After group discussion, 39 items deemed less relevant were removed at this stage (Supplementary Appendix 2).

A total of 20 THA patients participated in the individual concept elicitation interviews. All patients reported that the items were comprehensive and covered a broad range of concerns. In addition, nine patients specifically mentioned the issue of pain triggered by changes in weather. After discussion and consensus among the research team, we decided to add a new item at this stage: “Changes in weather make my pain more pronounced.”

A 5-level Likert scale was used for the response options, and a duration of 1 month was selected to ensure accurate values. Based on these revisions, a preliminary CTP containing 163 items was developed (Table 6).

**TABLE 6:** The degree of centralization and coordination of the importance scores of items in two rounds of Delphi expert consultation.

Items	First round		Second round		Whether to ultimately include it in the CTP and its corresponding dimension
	Important score	CV	Important score	CV	
F1. I can go up and down stairs at a normal pace.	4.65 ± 0.61	0.13	4.60 ± 0.63	0.14	Yes (Activities and functions)
F2. I can go up and down stairs without using handrails.	4.18 ± 0.73	0.17	3.73 ± 0.46	0.12	NO
F3. I can get in and out of the car by myself.	4.53 ± 0.72	0.16	4.60 ± 0.63	0.14	Yes (Activities and functions)
F4. I can go up or down slopes.	4.65 ± 0.49	0.11	4.60 ± 0.51	0.11	Yes (Activities and functions)
F5. I can travel alone using public transportation (bus, subway, etc.).	4.35 ± 0.70	0.16	4.67 ± 0.49	0.1	Yes (Activities and functions)
F6. I can step over items on the floor, such as toys, sports equipment, or small boxes.	4.88 ± 0.33	0.07	4.87 ± 0.35	0.07	Yes (Activities and functions)
F7. I can ride a bicycle outdoors.	4.76 ± 0.44	0.09	4.80 ± 0.41	0.09	Yes (Activities and functions)
F8. I can perform a squatting movement.	3.24 ± 1.03	0.32	–	–	No
F9. I can squat and then stand up.	4.24 ± 0.66	0.16	4.60 ± 0.51	0.11	Yes (Activities and functions)
F10. I can hop on one foot.	4.18 ± 0.53	0.13	4.27 ± 0.46	0.11	Yes (Activities and functions)
F11. I can straighten and bend my legs.	4.65 ± 0.49	0.11	4.67 ± 0.49	0.1	Yes (Activities and functions)
F12. I can walk fast for more than 10 minutes.	2.76 ± 1.15	0.41	–	–	No
F13. I can participate in swimming exercises.	2.59 ± 0.80	0.31	–	–	No
F14. I can run more than one kilometer.	4.29 ± 0.69	0.16	4.27 ± 0.70	0.16	Yes (Activities and functions)
F15. I can walk for more than half an hour.	4.47 ± 0.62	0.14	4.40 ± 0.63	0.14	Yes (Activities and functions)
F16. I can jog on flat or uneven surfaces.	3.41 ± 0.87	0.26	–	–	No
F17. I need to hold onto something when using the toilet (sitting down or standing up).	4.00 ± 0.71	0.18	3.80 ± 0.56	0.15	No
F18. I can only use the toilet while sitting.	3.47 ± 0.94	0.27	–	–	No
F19. I can use the toilet while squatting.	4.12 ± 0.70	0.17	4.07 ± 0.70	0.17	Yes (Activities and functions)
F20. I can complete some heavy household chores (moving heavy boxes, scrubbing the floor, etc.).	4.59 ± 0.51	0.11	4.53 ± 0.52	0.11	Yes (Activities and functions)
F21. I can complete some light household activities (sweeping, vacuuming, cleaning, etc.).	4.59 ± 0.51	0.11	4.53 ± 0.52	0.11	Yes (Activities and functions)
F22. I can perform gardening activities (weeding, watering, hoeing, etc.).	2.53 ± 0.62	0.25	–	–	No
F23. I can care for the elderly or children (pushing a wheelchair, pushing a stroller, lifting).	4.00 ± 0.35	0.09	4.20 ± 0.41	0.10	Yes (Activities and functions)
F24. I can stand in a stationary position with my eyes closed.	4.00 ± 0.79	0.2	3.73 ± 0.59	0.16	NO
F25. I can maintain a standing position.	4.41 ± 0.62	0.14	4.40 ± 0.63	0.14	Yes (Activities and functions)
F26. I can stand on one leg.	4.24 ± 0.75	0.18	4.20 ± 0.77	0.18	Yes (Activities and functions)
F27. I can twist my torso while standing.	4.06 ± 0.66	0.16	3.87 ± 0.52	0.13	No
F28. I cannot do work that requires standing or walking.	2.76 ± 0.83	0.3	–	–	No
F29. I can bend over and pick up items from the floor.	4.82 ± 0.39	0.08	4.80 ± 0.41	0.09	Yes (Activities and functions)
F30. When sitting on a low sofa, I need armrests or must sit down heavily.	4.29 ± 0.77	0.18	4.20 ± 0.77	0.18	Yes (Activities and functions)
F31. When getting up from a low sofa, I need to move forward first.	3.41 ± 0.87	0.26	–	–	No
F32. I feel uncomfortable if I remain seated for too long.	2.76 ± 0.90	0.33	–	–	No
F33. I can sit comfortably on an ordinary chair for more than an hour.	4.06 ± 0.66	0.16	4.07 ± 0.70	0.17	Yes (Activities and functions)
F34. I can turn over in bed.	4.65 ± 0.61	0.13	4.60 ± 0.63	0.14	Yes (Activities and functions)
F35. I can get up from bed by myself.	4.47 ± 0.72	0.16	4.53 ± 0.74	0.16	Yes (Activities and functions)
F36. I can put on and take off my shoes and stockings.	4.47 ± 0.62	0.14	4.40 ± 0.63	0.14	Yes (Activities and functions)
F37. I cannot cut my toenails by myself.	2.88 ± 1.11	0.39	–	–	NO
F38. I can take a bath and get dressed by myself.	4.35 ± 0.70	0.16	4.33 ± 0.72	0.17	Yes (Activities and functions)
P1. I feel pain when going up and down stairs.	4.76 ± 0.44	0.09	4.73 ± 0.46	0.10	Yes (Pain)
P2. I feel pain when walking on uneven surfaces.	4.00 ± 0.61	0.15	–	–	No
P3. The pain is more noticeable on hard surfaces.	4.06 ± 0.75	0.18	–	–	No
P4. I feel pain when walking on flat ground.	4.82 ± 0.39	0.08	4.8 ± 0.41	0.09	Yes (Pain)
P5. I feel pain when crossing obstacles.	4.24 ± 0.66	0.16	4.27 ± 0.70	0.16	Yes (Pain)
P6. I experience pain during vigorous activities.	4.00 ± 0.50	0.13	4.00 ± 0.53	0.13	Yes (Pain)
P7. I feel pain when squatting or standing up.	4.35 ± 0.79	0.18	4.33 ± 0.82	0.19	Yes (Pain)
P8. I feel pain when carrying heavy objects.	2.82 ± 0.88	0.31	–	–	No
P9. I experience pain while sitting.	4.06 ± 0.66	0.16	4.07 ± 0.70	0.17	Yes (Pain)
P10. I feel pain when getting up from a chair or the floor.	4.59 ± 0.51	0.11	4.53 ± 0.52	0.11	Yes (Pain)
P11. I feel pain when standing.	4.29 ± 0.59	0.14	4.27 ± 0.59	0.14	Yes (Pain)
P12. I experience pain when using a squat toilet.	4.76 ± 0.44	0.09	4.73 ± 0.46	0.10	Yes (Pain)
P13. I feel pain when using a sitting toilet.	4.00 ± 0.61	0.15	3.80 ± 0.56	0.15	No
P14. I feel pain when lying in bed at night.	4.65 ± 0.49	0.11	4.60 ± 0.51	0.11	Yes (Pain)
P15. Even while resting, I still feel pain.	4.47 ± 0.72	0.16	4.47 ± 0.74	0.17	Yes (Pain)
P16. I feel pain when fully extending my hip joint.	3.12 ± 0.99	0.32	–	–	No
P17. I feel pain when fully bending my hip joint.	3.12 ± 0.86	0.28	–	–	No
P18. I can feel pain in the affected area almost all the time.	4.59 ± 0.51	0.11	4.6 ± 0.51	0.11	Yes (Pain)
P19. The pain is more severe at night.	4.35 ± 0.61	0.14	4.33 ± 0.62	0.14	Yes (Pain)
P20. Changes in the weather make my pain more pronounced.	4.12 ± 0.60	0.15	3.87 ± 0.52	0.13	No
P21. My affected hip joint experiences sudden and severe pain (“stabbing pain,” “piercing pain,” or “spasm”).	4.35 ± 0.70	0.16	4.40 ± 0.74	0.17	Yes (Pain)
P22. It is difficult to fall asleep due to pain.	3.53 ± 1.07	0.30	–	–	No
P23. I wake up at night due to pain.	2.94 ± 1.03	0.35	–	–	No
S1. I feel swelling in the position of the hip joint.	4.53 ± 0.51	0.11	4.53 ± 0.52	0.11	Yes (Symptoms)
S2. I feel stiffness in the hip joint in the morning.	4.35 ± 0.70	0.16	4.27 ± 0.70	0.16	Yes (Symptoms)
S3. I feel stiffness in the hip joint when sitting or lying before going to bed at night.	4.59 ± 0.51	0.11	3.8 ± 0.77	0.20	No
S4. I feel a rubbing sound or can hear a clicking sound in the hip joint.	4.53 ± 0.51	0.11	4.53 ± 0.52	0.11	Yes (Symptoms)
S5. I often feel that my legs can’t support myself to stand or walk.	4.47 ± 0.51	0.12	4.47 ± 0.52	0.12	Yes (Symptoms)
S6. I limp when walking.	4.88 ± 0.33	0.07	4.87 ± 0.35	0.07	Yes (Symptoms)
S7. It’s difficult for me to separate my thighs largely.	4.35 ± 0.49	0.11	3.93 ± 0.59	0.15	No
S8. My legs have no strength.	4.06 ± 0.56	0.14	4.13 ± 0.52	0.12	Yes (Symptoms)
S9. I feel tired.	4.00 ± 0.35	0.09	3.73 ± 0.59	0.16	No
Q1. I often realize my hip joint problem.	4.53 ± 0.62	0.14	4.53 ± 0.64	0.14	Yes (QoL)
Q2. The hip joint problem makes me have no self-confidence.	4.35 ± 0.61	0.14	4.40 ± 0.63	0.14	Yes (QoL)
Q3. The problem of my hip joint has changed my lifestyle.	4.41 ± 0.62	0.14	4.47 ± 0.64	0.14	Yes (QoL)
Q4. The problem of the hip joint always distracts me.	4.00 ± 0.61	0.15	3.73 ± 0.70	0.19	No
Q5. The hip joint problem affects my work.	4.47 ± 0.51	0.12	4.47 ± 0.52	0.12	Yes (QoL)
Q6. I avoid talking about the joint problem.	4.18 ± 0.64	0.15	4.13 ± 0.64	0.15	Yes (QoL)
Q7. I dare not hold the child because of worrying about the problem of the hip joint.	4.06 ± 0.43	0.11	4.07 ± 0.46	0.11	Yes (QoL)
Q8. I always need others’ help in daily life.	4.12 ± 0.60	0.15	4.07 ± 0.59	0.15	Yes (QoL)
Q9. I worry about the side effects or sequelae of the treatment.	4.12 ± 0.60	0.15	4.07 ± 0.59	0.15	Yes (QoL)
Q10. I need to walk with crutches.	4.59 ± 0.51	0.11	4.53 ± 0.52	0.11	Yes (QoL)
Q11. It’s very difficult for me to take a bath and get dressed independently.	4.59 ± 0.62	0.13	4.53 ± 0.64	0.14	Yes (QoL)
Q12. My performance in recreational or sports activities has decreased a lot.	4.18 ± 0.53	0.13	3.67 ± 0.82	0.22	No
Q13. I often feel worried.	4.41 ± 0.62	0.14	–	–	No
Q14. I feel that I am older than my actual age.	2.35 ± 0.86	0.37	–	–	No
Q15. My family is not as happy as before.	4.18 ± 0.64	0.15	4.13 ± 0.64	0.15	Yes (QoL)
Q16. After the hip joint surgery, my quality of life has improved significantly.	4.41 ± 0.62	0.14	4.47 ± 0.64	0.14	Yes (QoL)
Q17. I can basically do the things I could do in the past now.	4.53 ± 0.62	0.14	4.47 ± 0.64	0.14	Yes (QoL)
Q18. I can go shopping alone.	2.82 ± 1.07	0.38	–	–	No
Q19. I can cross the road by myself.	2.29 ± 1.10	0.48	–	–	No
Q20. I can carry out daily leisure and recreational activities.	4.41 ± 0.51	0.11	4.47 ± 0.52	0.12	Yes (QoL)
Q21. My hip joint problem affects the quality of my sleep.	4.24 ± 0.66	0.16	4.27 ± 0.7	0.16	Yes (QoL)
Q22.Hip problems have affected the quality of my sex life.	4.00 ± 1.00	0.25	4.33 ± 0.98	0.23	Yes (QoL)
Q23. I haven’t had a full night’s sleep for a long time.	2.59 ± 0.87	0.34	–	–	No
Q24. My energy is obviously not as good as before.	2.06 ± 0.75	0.36	–	–	No
PS1. I can share happiness and sadness with my friends and family.	3.71 ± 0.59	0.16	–	–	No
PS2. I do feel that my friends and family listen to me.	3.00 ± 0.79	0.26	–	–	No
PS3. My friends and family help me with household chores.	4.06 ± 0.43	0.11	4.00 ± 0.38	0.09	Yes (Perceptionand social support)
PS4. My friends and family provide transportation services for me.	2.94 ± 0.90	0.31	–	–	No
PS5. My friends and family help me exercise.	4.53 ± 0.62	0.14	4.47 ± 0.64	0.14	Yes (Perceptionand social support)
PS6. When I’m sick, my friends and family are always with me.	4.41 ± 0.62	0.14	4.40 ± 0.63	0.14	Yes (Perceptionand social support)
PS7. I’m satisfied with the support from my family and friends.	4.00 ± 0.61	0.15	4.00 ± 0.65	0.16	Yes (Perceptionand social support)
PS8. I’m satisfied with my interpersonal relationships.	4.12 ± 0.49	0.12	3.93 ± 0.59	0.15	No
PS9. I’m satisfied with my working ability.	4.41 ± 0.62	0.14	4.33 ± 0.62	0.14	Yes (Perception and social support)
PS10. My economic condition can meet my needs.	4.24 ± 0.56	0.13	4.20 ± 0.56	0.13	Yes (Perception and social support)
PS11. I’m often afraid of becoming disabled.	4.41 ± 0.51	0.11	4.40 ± 0.51	0.12	Yes (Perception and social support)
PS12. I feel embarrassed when being seen.	4.41 ± 0.62	0.14	4.40 ± 0.63	0.14	Yes (Perception and social support)
PS13. I’m afraid of relying on others all the time.	4.35 ± 0.61	0.14	4.33 ± 0.62	0.14	Yes (Perception and social support)
PS14. I feel that I have become a burden to my family.	4.76 ± 0.44	0.09	4.73 ± 0.46	0.10	Yes (Perception and social support)
PS15. I’m embarrassed to ask for help actively.	4.47 ± 0.51	0.12	4.47 ± 0.52	0.12	Yes (Perception and social support)
M1. I feel nervous or “excited”:	2.88 ± 0.78	0.27	–	–	No
M2. Worried thoughts flash through my mind:	3.41 ± 0.71	0.21	–	–	No
M3. I feel happy:	2.29 ± 0.85	0.37	–	–	No
M4. Because of the hip joint disease, I sometimes get angry or feel nervous.	4.00 ± 0.61	0.15	4.07 ± 0.59	0.15	Yes (Mental)
M5. Because of the hip joint disease, I feel depressed and avoid going out.	4.47 ± 0.51	0.12	4.53 ± 0.52	0.11	Yes (Mental)
M6. I can sit easily and feel relaxed:	4.06 ± 0.66	0.16	3.87 ± 0.52	0.13	No
M7. I feel nervous about participating in sports.	4.41 ± 0.51	0.11	4.4 ± 0.51	0.12	Yes (Mental)
M8. I feel relaxed about participating in sports.	4.00 ± 0.71	0.18	3.93 ± 0.70	0.18	No
M9. I believe that my hip joint won’t have problems when participating in sports.	4.59 ± 0.51	0.11	4.53 ± 0.52	0.11	Yes (Mental)
M10. I believe that I can participate in sports without any concerns.	4.65 ± 0.49	0.11	–	–	No
M11. I believe that my hip joint can withstand the pressure.	4.59 ± 0.51	0.11	4.60 ± 0.51	0.11	Yes (Mental)
M12. I believe that I can return to my previous participation level in sports.	3.41 ± 0.87	0.26	–	–	No
M13. I think my life is meaningful.	2.76 ± 0.83	0.30	–	–	No
M14. I don’t have many negative emotions.	3.41 ± 0.71	0.21	–	–	No
M15. I feel very safe in daily life.	2.29 ± 0.85	0.37	–	–	No
M16. Because of the hip joint disease, I’m anxious about my livelihood/daily life.	4.53 ± 0.72	0.16	4.47 ± 0.74	0.17	Yes (Mental)
M17. Because of the hip joint disease, I’m dissatisfied with my health condition.	4.18 ± 0.53	0.13	4.27 ± 0.46	0.11	Yes (Mental)
M18. Because of the hip joint disease, it’s difficult for me to actively engage in various things.	4.29 ± 0.69	0.16	4.27 ± 0.7	0.16	Yes (Mental)
M19.I’m worried about problems with my hip joint.(Add)	–	–	4.47 ± 0.64	0.14	Yes (Mental)
E1. I hope my pain can be relieved.	4.88 ± 0.33	0.07	4.87 ± 0.35	0.07	Yes (Expectation)
E2. I hope to relieve the pain during the day.	2.24 ± 0.83	0.37	–	–	No
E3. I hope to relieve the pain that disturbs my sleep.	2.88 ± 0.78	0.27	–	–	No
E4. I hope to no longer need to take medicine.	4.24 ± 0.56	0.13	–	–	No
E5. I hope to improve my mental health.	2.88 ± 0.78	0.27	–	–	No
E6. I hope to no longer limp.	4.59 ± 0.71	0.16	4.60 ± 0.74	0.16	Yes (Expectation)
E7. I hope to improve my walking ability.	4.65 ± 0.49	0.11	3.80 ± 0.68	0.18	No
E8. I hope to no longer need crutches or other walking aids.	4.41 ± 0.51	0.11	4.40 ± 0.51	0.12	Yes (Expectation)
E9. I hope to improve my ability to go up and down stairs.	4.47 ± 0.62	0.14	4.47 ± 0.64	0.14	Yes (Expectation)
E10. I hope to improve my ability to get in and out of bed or a vehicle, and to sit in or rise from a chair or armchair.	4.35 ± 0.70	0.16	3.20 ± 0.68	0.45	No
E11. I hope to improve my ability to stand up and stand.	4.35 ± 0.61	0.14	3.93 ± 0.59	0.15	No
E12. I hope to improve my ability to carry out activities at home (e.g., doing housework, gardening, etc.).	4.12 ± 0.60	0.15	4.13 ± 0.64	0.15	Yes (Expectation)
E13. I hope to be able to improve my ability to do housework and yard work.	2.18 ± 0.73	0.33	–	–	No
E14. I hope to improve my ability to carry out activities outside the home (e.g., shopping, etc.).	4.41 ± 0.62	0.14	4.4 ± 0.63	0.14	Yes (Expectation)
E15. I hope to improve my ability to wear socks, stockings and shoes.	4.12 ± 0.60	0.15	4.13 ± 0.64	0.15	Yes (Expectation)
E16. I hope to improve my ability to cut my toenails.	2.88 ± 0.70	0.24	–	–	No
E17. I hope to improve my ability to carry out physical or sports activities.	4.47 ± 0.62	0.14	4.4 ± 0.63	0.14	Yes (Expectation)
E18. I hope to improve my ability to engage in recreational activities.	4.18 ± 0.73	0.17	3.93 ± 0.80	0.2	No
E19. I hope to engage in occupational activities.	4.53 ± 0.62	0.14	4.47 ± 0.64	0.14	Yes (Expectation)
Sa1. Compared with before the surgery, the pain when I walk has been relieved a lot.	4.00 ± 0.61	0.15	–	–	No
Sa2. After the surgery, my pain has been relieved.	4.71 ± 0.47	0.1	4.80 ± 0.41	0.09	Yes (Satisfaction)
Sa3. Compared with before the surgery, my joint hardly hurts today.	3.00 ± 0.50	0.17	–	–	No
Sa4. Compared with before the surgery, I basically don’t need to take painkillers anymore.	4.24 ± 0.56	0.13	4.27 ± 0.59	0.14	Yes (Satisfaction)
Sa5. Compared with before the surgery, the pain when I try to fall asleep has been reduced a lot.	4.24 ± 0.66	0.16	3.87 ± 0.74	0.19	No
Sa6. Compared with before the surgery, the pain at night has been relieved a lot.	4.35 ± 0.61	0.14	4.33 ± 0.62	0.14	Yes (Satisfaction)
Sa7. Compared with before the surgery, I can sleep through the night now.	4.12 ± 0.33	0.08	–	–	No
Sa8. My sleep quality has been greatly improved.	4.35 ± 0.61	0.14	4.33 ± 0.62	0.14	Yes (Satisfaction)
Sa9. Compared with before the surgery, the pain when I get into bed has been relieved a lot.	4.00 ± 0.61	0.15	4.00 ± 0.65	0.16	Yes (Satisfaction)
Sa10. Compared with before the surgery, my muscle spasms are almost gone.	4.00 ± 0.79	0.20	4.27 ± 0.46	0.11	Yes (Satisfaction)
Sa11. Compared with before the surgery, my appetite has improved a lot.	2.29 ± 0.85	0.37	–	–	No
Sa12. Compared with before the surgery, my mood has improved a lot.	4.06 ± 0.56	0.14	4.13 ± 0.52	0.12	Yes (Satisfaction)
Sa13. Compared with before the surgery, my energy level has increased a lot.	4.00 ± 0.35	0.09	3.73 ± 0.59	0.16	No
Sa14. Compared with before the surgery, I feel generally very well.	4.53 ± 0.62	0.14	4.53 ± 0.64	0.14	Yes (Satisfaction)
Sa15. I am very satisfied with the surgical outcome.	4.35 ± 0.61	0.14	4.40 ± 0.63	0.14	Yes (Satisfaction)
Sa16. After the surgery, my ability to carry out daily activities has improved.	4.41 ± 0.62	0.14	4.47 ± 0.64	0.14	Yes (Satisfaction)
Sa17. After the surgery, my ability to carry out recreational activities has improved.	4.00 ± 0.79	0.2	–	–	No

A total of 20 questionnaires were distributed in the first round of the Delphi method, and 17 valid responses were collected, resulting in a valid response rate of 85.0%. Of the 17 experts, 15 (88.2%) proposed 29 modifications (Supplementary Appendix 3). In the second round, 17 questionnaires were again distributed, and 15 valid responses were collected, yielding an effective response rate of 88.2%. Two experts from Round 1 did not participate in Round 2 due to non-response; the overall disciplinary composition of the panel remained similar (Table 4), but the potential for non-response bias is acknowledged in the Limitations section.

The expert judgment awareness values for the two rounds of the Delphi method were 0.91 ± 0.07 and 0.92 ± 0.07 (Table 2), respectively, while the coefficient of expert judgment skill values were 0.92 ± 0.10 and 0.93 ± 0.10 (Table 3). The coefficient of expert authority values for the two rounds were 0.91 ± 0.08 and 0.93 ± 0.08. All of these values were above 0.7, indicating that the consultation results were reliable.

In this study, the importance scores obtained from the two rounds of Delphi consultation ranged from 2.06 to 4.88 in the first round and from 2.87 to 4.87 in the second round. The coefficient of variation (CV) of the importance scores was between 0.07 and 0.48 in the first round and between 0.07 and 0.45 in the second round (Table 6). Kendall’s W for the two rounds was 0.469 and 0.223, respectively (both *P* < 0.001), indicating significant agreement among experts (Round 1: χ^2^ = 1299.60, *df* = 163; Round 2: χ^2^ = 394.71, *df* = 118). The smaller CV range in the second round suggests that expert opinions became more aligned after iterative feedback.

Based on participant feedback, no adjustments to the dimensions were necessary. According to the screening criteria, 65 items were either merged or deleted, and one item was added. Additionally, 21 items were modified to enhance readability and clarity. The resulting preliminary CTP consists of eight dimensions and 99 items; the dimension structure and example items are summarized in Table 7, and the full item pool with Delphi ratings is presented in Table 6. After the Delphi consultation, the research team convened to discuss and propose scoring rules for each item.

**TABLE 7:** Summary of the eight dimensions of the CTP with the number of items and example items.

Dimension	No. of items	Example items (from final CTP)
Activities and functions	24	F1. I can go up and down stairs at a normal pace. F15. I can walk for more than half an hour. F38. I can take a bath and get dressed by myself.
Pain	14	P4. I feel pain when walking on flat ground. P14. I feel pain when lying in bed at night. P15. Even while resting, I still feel pain.
Symptoms	6	S2. I feel stiffness in the hip joint in the morning. S6. I limp when walking. S8. My legs have no strength.
Quality of life	16	Q3. The problem of my hip joint has changed my lifestyle. Q5. The hip joint problem affects my work. Q21. My hip joint problem affects the quality of my sleep.
Perception and social support	11	PS3. My friends and family help me with household chores. PS7. I’m satisfied with the support from my family and friends. PS14. I feel that I have become a burden to my family.
Mental	9	M5. Because of the hip joint disease, I feel depressed and avoid going out. M16. Because of the hip joint disease, I’m anxious about my livelihood/daily life. M17. Because of the hip joint disease, I’m dissatisfied with my health condition.
Expectation	9	E1. I hope my pain can be relieved. E8. I hope to no longer need crutches or other walking aids. E9. I hope to improve my ability to go up and down stairs.
Satisfaction	10	Sa2. After the surgery, my pain has been relieved. Sa15. I am very satisfied with the surgical outcome. Sa16. After the surgery, my ability to carry out daily activities has improved.

### 3.1 Patient cognitive debriefing

Twenty patients after THA underwent think-aloud interviews to assess the relevance, comprehensibility, and comprehensiveness of the questionnaire items. Overall, the patients considered the questionnaire easy to understand and that it covered the core concerns post-surgery. Twelve of the 20 patients reported wording imperfections in some items and requested supplementary examples. The items were revised accordingly to enhance clarity and cultural appropriateness. For instance, the original item “I can step over items on the floor” was revised to “I can step over items on the floor, such as toys, sports equipment, or small boxes” because patients reported that the specific referent of “items” was unclear. Similarly, the original item “I can complete some heavy household chores” was modified to “I can complete some heavy household chores (moving heavy boxes, scrubbing the floor, etc.)” as patients noted that the definition of “heavy household chores” was ambiguous. A total of 16 items were revised based on the feedback from the cognitive interviews and item-level revisions. Detailed information is provided in Supplementary Appendix 4.

## 4 Discussion

### 4.1 Summary of main findings

The biopsychosocial medical model represents a significant paradigm shift in the medical field (64). Its core principle lies in moving beyond the single perspective of the traditional biomedical model to view health and disease as the result of the combined influence of biological, psychological, and social factors. PROMs are important tools in healthcare for capturing the perceptions of patients of their health status, functional capacity, and quality of life, as well as for assessing subjective outcomes such as pain levels, physical function, and emotional wellbeing (65). In surgical fields like orthopedics, patient satisfaction and functional recovery are central to evaluating treatment effects (66). Therefore, this study adopts the biopsychosocial medical model as the theoretical basis for developing the theoretical framework of the PROMs for THA. THA not only involves the repair of physiological structures but also affects the psychological states (such as anxiety and depressive emotions) and social functions (such as social activities and family roles) of patients. (67). By using this framework to construct a conceptual model, the final conceptual model consists of 8 dimensions, comprising a total of 99 items. We can comprehensively address multiple aspects, including the physiological recovery, psychological wellbeing, and social adaptation of patients after THA, thereby enabling the scale to more accurately reflect the real experiences and outcomes of patients.

### 4.2 Comparison with existing THA PROMs

However, global standards must be adapted to the unique context of each country, and addressing cross-cultural differences is a critical challenge in this process. PROMs developed for THA patients within Western cultural contexts may not fully align with the needs of Chinese patients. Tsinghua University used the Evaluation of Patient-Reported Outcomes (EMPRO) tool to qualitatively and quantitatively assess six THA PROMs suitable for Chinese patients, including the Oxford Hip Score (OHS), the Simplified Chinese version of the International Hip Outcome Tool-33 (SC-iHOT-33), and the Hip Disability and Osteoarthritis Outcome Score (HOOS). The results showed that none of these scales met the threshold for clinically acceptable application. A study by Yanqiong et al. (68) found that Chinese patients scored higher in domains such as symptoms, pain, stiffness, and daily activities but lower in sports/recreational activities and quality of life compared to patients in Western countries. This discrepancy may stem from differences in healthcare technology, regional culture, and the emphasis placed by foreign healthcare organizations on post-discharge continuity of care.

Prominent instruments such as HOOS, OHS, and iHOT-33 capture important postoperative outcomes but differ in conceptual emphasis. HOOS focuses on five domains (pain, symptoms, function in daily living, sport/recreation function, and hip-related quality of life), and OHS is a brief 12-item tool primarily reflecting pain and function. iHOT-33 includes domains spanning symptoms/functional limitations, sports/recreation, job-related concerns, and social/emotional/lifestyle concerns. In contrast, while the CTP retains core domains that overlap with these measures (function, pain, symptoms, and quality of life), it additionally includes dedicated dimensions for cognitive and social support, expectations, and satisfaction. These constructs were repeatedly highlighted by Chinese patients and clinicians during concept elicitation and are particularly relevant in an elective surgery context and within a family-oriented sociocultural environment.

Among the commonly used PROMs for THA patients, certain items appear less relevant to Chinese patients. For instance, activities such as gardening, entering/exiting a bathtub, golf, and bowling mentioned in HOOS, Forgotten Joint Score-12 (FJS-12), Hospital for Special Surgery (HSS) scale (56), and Yale Physical Activity Survey (YPAS) are culturally uncommon in China. Additionally, sensitive topics such as sexual activity frequency and quality (e.g., in iHOT-33, Osteo Arthritis of the Knee Hip Quality Of Life (OAKHQOL), and HSS) often result in low response rates among Chinese patients. A preliminary survey conducted by our group confirmed this issue: only about 10% of patients responded to the item “quality of sexual life,” and most of them chose the lowest response category (“rarely”).

From a measurement perspective, the Cronbach’s α values of the Non-Arthritic Hip Score (NAHS) and Modified Harris Hip Score (mHHS) fall below 0.8 (69–71), indicating potential limitations in internal consistency. Furthermore, the NAHS and International Hip Outcome Tool-33 (iHOT-33) were designed for younger patient populations and fail to adequately address the needs of elderly patients. The factor structure of the HOOS may exhibit instability in THA patients, particularly when assessing high-functioning individuals (72). Moreover, both the HOOS and OHS are prone to ceiling effects in patients with strong functional recovery, which limits their ability to differentiate outcomes in this subgroup (73, 74).

Currently, China lacks a PROM specifically designed for THA patients. Therefore, developing a CTP that reflects China’s sociocultural context, incorporates psychological factors, and is practical for use in Chinese healthcare settings is crucial. In this study, we conducted a series of in-depth interviews and utilized the Delphi method to establish the conceptual framework and preliminary content of the CTP. By integrating the perspectives of clinicians and patients, we laid a strong foundation for the formal development of the CTP.

### 4.3 Strengths of the study

Patel et al. (75) emphasized that the future of PROMs in clinical practice depends on standardization, personalization, and integration with longitudinal studies, with personalized assessment being a key focus. Tailoring PROMs to the characteristics of specific patient populations enhances the relevance and accuracy of measurements. This customization ensures that assessments reflect the unique needs of patients, ultimately improving satisfaction with care. Accordingly, the CTP incorporates distinct features of Chinese patients within its functional and quality of life dimensions.

The study (76) revealed that the age distribution of THA patients in China follows a unimodal pattern, with individuals aged 51–70 years constituting the predominant group. Notably, the 51–60 age range represents the peak incidence cohort, and this demographic demonstrates significantly higher preoperative comorbidity indices compared to younger patients. Compared with patients in other countries, Chinese patients scored higher on symptoms, pain, stiffness, and activities of daily living, but lower on sports/recreational activities and quality of life. This suggests that Chinese patients prioritize symptom alleviation and pain reduction over maintaining the ability to perform activities of daily living. Through multiple rounds of interviews and expert consultations, this study not only established symptom- and pain-related dimensions but also introduced culturally competent items in the domains of functionality and quality of life.

The pilot surveys revealed that elderly patients often had limited education and struggled to understand certain questions. Through iterative discussions with patients and experts, we revised ambiguous items using colloquial language and relatable examples. This approach improved clarity and enabled patients to respond more accurately.

This study emphasizes the importance of incorporating qualitative research methods and patient perspectives during scale development (77). Through concept elicitation, in-depth interviews were conducted to gain a comprehensive understanding of the descriptions of symptoms, disease impacts, and related experiences of patients. By prioritizing patient perspectives, we ensured that the voices and lived experiences of patients at the center of CTP development. Multiple rounds of patient feedback further optimized the language of scale items, making them more understandable, culturally appropriate, and user-friendly for Chinese patients.

In addition to patient input, experts from various disciplines were actively consulted to incorporate a range of professional insights. They noted that Chinese patients, particularly older adults, prioritize familial roles. As a result, we reduced items related to high-intensity sports or recreational activities in the functional and quality-of-life dimensions and added items such as “taking care of grandchildren,” “housework,” and “light physical labor” to better reflect the cultural emphasis on family-oriented values.

The foundational framework and preliminary elements of the CTP were established by reviewing and analyzing existing literature, supplemented by a series of qualitative studies and Delphi interviews. The CTP highlights the key priorities and issues facing Chinese THA patients and healthcare professionals throughout the perioperative period. This effort marks a significant milestone in the development of a finalized CTP designed specifically to address the unique needs of THA patients in China.

### 4.4 Limitations and future directions

Several limitations should be acknowledged. First, as all samples were collected from a single center (Xi’an Honghui Hospital), the generalizability of the findings to other regions and healthcare settings may be limited. To address this, a multi-center validation study is planned, enrolling THA patients with diverse demographics (e.g., age, education, economic status, comorbidities) and at different postoperative stages across multiple regions. This large-scale, multi-regional validation will systematically assess the reliability, validity, and applicability of the scale across varied populations and clinical settings, supporting its standardized use in Chinese clinical practice. Second, the present output is a preliminary 99-item pool; its length may reduce feasibility in routine practice and therefore requires future item reduction and comprehensive psychometric testing (e.g., structural validity, reliability, measurement error, responsiveness, and interpretability) in larger multicenter samples. Third, although patients were extensively involved in concept elicitation and cognitive debriefing, they did not participate in the Delphi importance scoring, which may have resulted in greater weighting of expert perspectives during item prioritization. Fourth, a modest attrition between Delphi rounds (17–15 experts) may introduce potential non-response bias.

However, scale development is a dynamic process of continuous optimization and validation. To further enhance the quality and applicability of the scale, subsequent research will conduct multiple rounds of large-sample clinical surveys and multi-center empirical studies. Item selection methods guided by Classical Test Theory will be comprehensively applied to systematically evaluate the psychometric properties of each item. Specifically, exploratory factor analysis and confirmatory factor analysis will be combined to optimize the scale’s factor structure and improve model fit. Concurrently, the Item Response Theory model will be introduced to assess item discrimination and difficulty parameters, progressively eliminating items with poor psychometric performance, with the goal of refining the scale to a reasonable length of approximately 30 items. Subsequently, statistical methods such as reliability and validity testing will be employed to comprehensively evaluate the psychometric properties of the refined scale, ultimately resulting in a PROM for THA suitable for clinical practice in China.

## Data Availability

The datasets presented in this study can be found in online repositories. The names of the repository/repositories and accession number(s) can be found below: The dataset used in this study is available from the Mendeley data. Accessible via Mendeley Data, V1, doi: 10.17632/tm734vfj5r.1.

## References

[1] Grayson CW Decker RC. Total joint arthroplasty for persons with osteoarthritis. *PM R.* (2012) 4(5 Suppl):S97–103. 10.1016/j.pmrj.2012.02.018 22632709

[2] Joint Surgery Group, Orthopaedic Branch of Chinese Medical Association, Osteoarthritis Group, Orthopaedic Surgeons Branch of Chinese Medical Doctor Association, National Clinical Research Center for Geriatric Diseases,. Chinese guidelines for the diagnosis and treatment of osteoarthritis (2021 edition). *Chin J Orthopaedics.* (2021) 41:1291–314.

[3] Li J Gao X Dou T Gao Y Li X Zhu W. Quantitative evaluation of GPT-4’s performance on US and Chinese osteoarthritis treatment guideline interpretation and orthopaedic case consultation. *BMJ Open.* (2024) 14:e082344. 10.1136/bmjopen-2023-082344 39806703 PMC11749315

[4] Sarhan O Megalla M Imam N Ren AN Redfern RE Klein GR. Improved patient reported outcomes with the direct anterior approach versus the posterior approach for total hip arthroplasty in the early post-operative period. *Arch Orthop Trauma Surg.* (2024) 144:2373–80. 10.1007/s00402-024-05271-z 38520548

[5] Hamilton DF Giesinger JM Giesinger K. It is merely subjective opinion that patient-reported outcome measures are not objective tools. *Bone Joint Res.* (2017) 6:665–6. 10.1302/2046-3758.612.BJR-2017-0347 29212762 PMC5935812

[6] Ngwayi JRM Tan J Liang N Sita EGE Obie KU Porter DE. Systematic review and standardised assessment of Chinese cross-cultural adapted hip Patient Reported Outcome Measures (PROMs). *PLoS One.* (2021) 16:e0257081. 10.1371/journal.pone.0257081 34543314 PMC8452074

[7] Pacheco-Brousseau L Stacey D Desmeules F Ben Amor S Dervin G Beaulé PE et al. Determining appropriateness of total joint arthroplasty for hip and knee osteoarthritis: a patient-centred conceptual model. *Musculoskeletal Care.* (2024) 22:e1927. 10.1002/msc.1927 39123311

[8] Zhang J Ye P Yang M Wu X Webster R Ivers R et al. Development of a conceptual framework to scale up co-managed care for older patients with hip fracture in China: a qualitative study. *BMC Health Serv Res.* (2023) 23:898. 10.1186/s12913-023-09910-w 37612703 PMC10463518

[9] Thomas JA Benson J Davidson P Ward PR. Opioids and the challenges of managing chronic non-cancer pain in rural Australia: a qualitative study. *Med J Aust.* (2025) 223:467–72. 10.5694/mja2.70022 40827117 PMC12579919

[10] Hu Y Jia LY Zheng SN. *COSMIN Study Design Checklist: For Patient-Reported Outcome Measures.* (2019). Available online at: https://www.cosmin.nl (accessed April 29, 2024).

[11] Jünger S Payne SA Brine J Radbruch L Brearley SG. Guidance on Conducting and REporting DElphi Studies (CREDES) in palliative care: recommendations based on a methodological systematic review. *Palliat Med.* (2017) 31:684–706. 10.1177/0269216317690685 28190381

[12] Yin Y Gao W Cui X Tang W. Development and validation of the Chinese patient-centered integrated care scale. *BMC Health Serv Res.* (2024) 24:1668. 10.1186/s12913-024-12156-9 39736739 PMC11687146

[13] Noushad B Van Gerven PWM de Bruin ABH. Twelve tips for applying the think-aloud method to capture cognitive processes. *Med Teach.* (2024) 46:892–7. 10.1080/0142159X.2023.2289847 38071621

[14] Shariff NJ. Utilizing the delphi survey approach: a review. *J Nurs Care.* (2015) 4:1–6. 10.4172/2167-1168.1000246

[15] Dai F Wei K Chen Y Ju M. Construction of an index system for qualitative evaluation of undergraduate nursing students innovative ability: a Delphi study. *J Clin Nurs.* (2019) 28:4379–88. 10.1111/jocn.15020 31411352

[16] Cui C Meng K. Development of an index system for evaluating the organisational capabilities of primary medical institutions: a modified Delphi study in China. *BMJ Open.* (2021) 11:e055422. 10.1136/bmjopen-2021-055422 34921088 PMC8689195

[17] Major MJ Fatone S Roth EJ. Validity and reliability of the Berg Balance Scale for community-dwelling persons with lower-limb amputation. *Arch Phys Med Rehabil.* (2013) 94:2194–202. 10.1016/j.apmr.2013.07.002 23856150

[18] Talbot S Hooper G Stokes A Zordan R. Use of a new high-activity arthroplasty score to assess function of young patients with total hip or knee arthroplasty. *J Arthroplasty.* (2010) 25:268–73. 10.1016/j.arth.2008.09.019 19056232

[19] Amstutz HC Thomas BJ Jinnah R Kim W Grogan T Yale C. Treatment of primary osteoarthritis of the hip. A comparison of total joint and surface replacement arthroplasty. *J Bone Joint Surg Am.* (1984) 66:228–41. 6693450

[20] Harwood RH Ebrahim S. The validity, reliability and responsiveness of the Nottingham Extended Activities of Daily Living scale in patients undergoing total hip replacement. *Disabil Rehabil.* (2002) 24:371–7. 10.1080/10.1080/09638280110101541 12022787

[21] Katz JN Phillips CB Poss R Harrast JJ Fossel AH Liang MH et al. The validity and reliability of a total hip arthroplasty outcome evaluation questionnaire. *J Bone Joint Surg Am.* (1995) 77:1528–34. 10.2106/00004623-199510000-00007 7593061

[22] Mahomed N Gandhi R Daltroy L Katz JN. The self-administered patient satisfaction scale for primary hip and knee arthroplasty. *Arthritis.* (2011) 2011:591253. 10.1155/2011/591253 22046521 PMC3199955

[23] Turner-Stokes L Disler R Williams H. The rehabilitation complexity scale: a simple, practical tool to identify ‘complex specialised’ services in neurological rehabilitation. *Clin Med.* (2007) 7:593–9. 10.7861/clinmedicine.7-6-593 18193708 PMC4954366

[24] Hays RD Sherbourne CD Mazel RM. The RAND 36-item health survey 1.0. *Health Econ.* (1993) 2:217–27. 10.1002/hec.4730020305 8275167

[25] Buysse DJ Reynolds CF III Monk TH Berman SR Kupfer DJ. The pittsburgh sleep quality index: a new instrument for psychiatric practice and research. *Psychiatry Res.* (1989) 28:193–213. 10.1016/0165-1781(89)90047-4 2748771

[26] Gibbons E Hewitson P Morley D Jenkinson C Fitzpatrick R. The outcomes and experiences questionnaire: development and validation. *Patient Relat Outcome Meas.* (2015) 6:179–89. 10.2147/PROM.S82784 26213480 PMC4509457

[27] Zigmond AS Snaith RP. The hospital anxiety and depression scale. *Acta Psychiatr Scand.* (1983) 67:361–70. 10.1111/j.1600-0447.1983.tb09716.x 6880820

[28] Kortte KB Falk LD Castillo RC Johnson-Greene D Wegener ST. The hopkins rehabilitation engagement rating scale: development and psychometric properties. *Arch Phys Med Rehabil.* (2007) 88:877–84. 10.1016/j.apmr.2007.03.030 17601468

[29] Mohtadi NG Griffin DR Pedersen ME Chan D Safran MR Parsons N et al. The Development and validation of a self-administered quality-of-life outcome measure for young, active patients with symptomatic hip disease: the International Hip Outcome Tool (iHOT-33). *Arthroscopy.* (2012) 28:595–10.e1. 10.1016/j.arthro.2012.03.013. 22542433

[30] Wollmerstedt N Nöth U Ince A Ackermann H Martell JM Hendrich C. The daily activity questionnaire: a novel questionnaire to assess patient activity after total hip arthroplasty. *J Arthroplasty.* (2010) 25:475–80.e3. 10.1016/j.arth.2009.01.005. 19232888

[31] Mercer SW Maxwell M Heaney D Watt GC. The consultation and relational empathy (CARE) measure: development and preliminary validation and reliability of an empathy-based consultation process measure. *Fam Pract.* (2004) 21:699–705. 10.1093/fampra/cmh621 15528286

[32] Poquet N Lin C. The Brief Pain Inventory (BPI). *J Physiother.* (2016) 62:52. 10.1016/j.jphys.2015.07.001 26303366

[33] Behrend H Giesinger K Giesinger JM Kuster MS. The “forgotten joint” as the ultimate goal in joint arthroplasty: validation of a new patient-reported outcome measure. *J Arthroplasty.* (2012) 27:430–6.e1. 10.1016/j.arth.2011.06.035. 22000572

[34] Henkus HE Van Kampen PM Van Der Linden MH Hogervorst T. SUSHI: the Super Simple Hip score for younger patients. *Hip Int.* (2011) 21:361–6. 10.5301/HIP.2011.8399 21698589

[35] Swiontkowski MF Engelberg R Martin DP Agel J. Short musculoskeletal function assessment questionnaire: validity, reliability, and responsiveness. *J Bone Joint Surg Am.* (1999) 81:1245–60. 10.2106/00004623-199909000-00006 10505521

[36] Lee KA. Self-reported sleep disturbances in employed women. *Sleep.* (1992) 15:493–8. 10.1093/sleep/15.6.493 1475563

[37] Casartelli NC Bolszak S Impellizzeri FM Maffiuletti NA. Reproducibility and validity of the physical activity scale for the elderly (PASE) questionnaire in patients after total hip arthroplasty. *Phys Ther.* (2015) 95:86–94. 10.2522/ptj.20130557 25147185

[38] Yeung TS Wessel J Stratford P Macdermid J. Reliability, validity, and responsiveness of the lower extremity functional scale for inpatients of an orthopaedic rehabilitation ward. *J Orthop Sports Phys Ther.* (2009) 39:468–77. 10.2519/jospt.2009.2971 19487822

[39] Kuribayashi M Takahashi KA Fujioka M Ueshima K Inoue S Kubo T. Reliability and validity of the Japanese orthopaedic association hip score. *J Orthop Sci.* (2010) 15:452–8. 10.1007/s00776-010-1490-0 20721711

[40] Benedetti MG Franchignoni F Morri M Franchini N Natali E Giordano A. Rasch analysis of the Iowa Level of Assistance Scale in patients with total hip and knee arthroplasty. *Int J Rehabil Res.* (2014) 37:118–24. 10.1097/MRR.0000000000000043 24406302

[41] Dawson J Fitzpatrick R Carr A Murray D. Questionnaire on the perceptions of patients about total hip replacement. *J Bone Joint Surg Br.* (1996) 78:185–90. 8666621

[42] Wörner T Thorborg K Webster KE Stålman A Eek F. Psychological readiness is related to return to sport following hip arthroscopy and can be assessed by the Hip-Return to Sport after Injury scale (Hip-RSI). *Knee Surg Sports Traumatol Arthrosc.* (2021) 29:1353–61. 10.1007/s00167-020-06157-4 32699920 PMC8038984

[43] Mancuso CA Ranawat AS Meftah M Koob TW Ranawat CS. Properties of the patient administered questionnaires: new scales measuring physical and psychological symptoms of hip and knee disorders. *J Arthroplasty.* (2012) 27:575–82.e6. 10.1016/j.arth.2011.07.014. 21945079

[44] Bauman A Ainsworth BE Bull F Craig CL Hagströmer M Sallis JF et al. Progress and pitfalls in the use of the International Physical Activity Questionnaire (IPAQ) for adult physical activity surveillance. *J Phys Act Health.* (2009) 6(Suppl 1):S5–8. 10.1123/jpah.6.s1.s5 19998844

[45] Duckworth J Matar HE Divecha H Wynn Jones H Board TN. Preoperative pain catastrophisation may predict worse patient-reported outcomes after primary hip arthroplasty: a pilot study. *J Orthop.* (2020) 20:186–9. 10.1016/j.jor.2020.01.025 32025146 PMC6997509

[46] Naal FD Impellizzeri FM Wasmer M Mannion AF Leunig M. Schulthess Hip Score (5 items) for assessing disability in patients undergoing total hip arthroplasty. *Dev Validat. Orthopade.* (2010) 39:834–41. 10.1007/s00132-010-1611-7 20383491

[47] Dolan P. Modeling valuations for EuroQol health states. *Med Care.* (1997) 35:1095–108. 10.1097/00005650-199711000-00002 9366889

[48] Peters LL Boter H Buskens E Slaets JP. Measurement properties of the Groningen Frailty Indicator in home-dwelling and institutionalized elderly people. *J Am Med Dir Assoc.* (2012) 13:546–51. 10.1016/j.jamda.2012.04.007 22579590

[49] Nilsdotter AK Lohmander LS Klässbo M Roos EM. Hip disability and osteoarthritis outcome score (HOOS)–validity and responsiveness in total hip replacement. *BMC Musculoskelet Disord.* (2003) 4:10. 10.1186/1471-2474-4-10 12777182 PMC161815

[50] van den Akker-Scheek I Stevens M Spriensma A van Horn JR. Groningen orthopaedic social support scale: validity and reliability. *J Adv Nurs.* (2004) 47:57–63. 10.1111/j.1365-2648.2004.03065.x 15186468

[51] Grafton K Foster N Wright C. Evaluation of the test-retest reliability of the short-form McGill pain questionnaire. *Physiotherapy.* (2002) 88:108–108.

[52] Snell DL Siegert RJ Surgenor LJ Dunn JA Hooper GJ. Evaluating quality of life outcomes following joint replacement: psychometric evaluation of a short form of the WHOQOL-Bref. *Qual Life Res.* (2016) 25:51–61. 10.1007/s11136-015-1044-1 26068734

[53] Pacheco-Brousseau L Poitras S Savard J Savard J Varin D Moreau G et al. Development of the French-Canadian Version of the Self-Administered Comorbidities Questionnaire (SCQ) in a hospital population undergoing hip or knee arthroplasty. *Orthop Traumatol Surg Res.* (2020) 106:557–61. 10.1016/j.otsr.2019.12.022 32265177

[54] Strickland LH Murray DW Pandit HG Jenkinson C. Development of a patient-reported outcome measure (PROM) and change measure for use in early recovery following hip or knee replacement. *J Patient Rep Outcomes.* (2020) 4:91. 10.1186/s41687-020-00262-1 33159610 PMC7648815

[55] Rat AC Pouchot J Coste J Baumann C Spitz E Retel-Rude N et al. Development and testing of a specific quality-of-life questionnaire for knee and hip osteoarthritis: OAKHQOL (OsteoArthritis of Knee Hip Quality Of Life). *Joint Bone Spine.* (2006) 73:697–704. 10.1016/j.jbspin.2006.01.027 17126060

[56] Neuprez A Delcour JP Fatemi F Gillet P Mawet M François G et al. Development and validation of the French version of a tool assessing patient’s expectations in lower limb osteoarthritis. *J Orthop.* (2014) 12:46–57. 10.1016/j.jor.2014.06.002 25829752 PMC4354649

[57] Terwee CB Coopmans C Peter WF Roorda LD Poolman RW Scholtes VA et al. Development and validation of the computer-administered animated activity questionnaire to measure physical functioning of patients with hip or knee osteoarthritis. *Phys Ther.* (2014) 94:251–61. 10.2522/ptj.20120472 24029297

[58] Katz JN Perez MT Niu NN Dong Y Brownlee SA Elman SA et al. Development and validation of a Spanish translation of the Yale activity questionnaire. *BMC Musculoskelet Disord.* (2014) 15:120. 10.1186/1471-2474-15-120 24708590 PMC4027998

[59] Saleh KJ Mulhall KJ Bershadsky B Ghomrawi HM White LE Buyea CM et al. Development and validation of a lower-extremity activity scale. Use for patients treated with revision total knee arthroplasty. *J Bone Joint Surg Am.* (2005) 87:1985–94. 10.2106/JBJS.D.02564 16140813

[60] Hill JC Kang S Benedetto E Blackburn S Smith S Dunn KM et al. Development and initial cohort validation of the Arthritis Research UK Musculoskeletal Health Questionnaire (MSK-HQ) for use across musculoskeletal care pathways. *BMJ Open.* (2016) 6:e012331. 10.1136/bmjopen-2016-012331 27496243 PMC4985936

[61] Goodman SM Mehta BY Kahlenberg CA Krell EC Nguyen J Finik J et al. Assessment of a satisfaction measure for use after primary total joint arthroplasty. *J Arthroplasty.* (2020) 35:1792–99.e4. 10.1016/j.arth.2020.02.039. 32173615

[62] Domzalski T Cook C Attarian DE Kelley SS Bolognesi MP Vail TP. Activity scale for arthroplasty patients after total hip arthroplasty. *J Arthroplasty.* (2010) 25:152–7. 10.1016/j.arth.2008.11.009 19106027

[63] Bellamy N Buchanan WW. A preliminary evaluation of the dimensionality and clinical importance of pain and disability in osteoarthritis of the hip and knee. *Clin Rheumatol.* (1986) 5:231–41. 10.1007/BF02032362 3731718

[64] Borrell-Carrió F Suchman AL Epstein RM. The biopsychosocial model 25 years later: principles, practice, and scientific inquiry. *Ann Fam Med.* (2004) 2:576–82. 10.1370/afm.245 15576544 PMC1466742

[65] Weldring T Smith SM. Patient-Reported Outcomes (PROs) and patient-reported outcome measures (PROMs). *Health Serv Insights.* (2013) 6:61–8. 10.4137/HSI.S11093 25114561 PMC4089835

[66] Golinelli D Grassi A Tedesco D Sanmarchi F Rosa S Rucci P et al. Patient reported outcomes measures (PROMs) trajectories after elective hip arthroplasty: a latent class and growth mixture analysis. *J Patient Rep Outcomes.* (2022) 6:95. 10.1186/s41687-022-00503-5 36085337 PMC9462642

[67] Lan RH Bell JW Samuel LT Kamath AF. Outcome measures in total hip arthroplasty: Have our metrics changed over 15 years? *Arch Orthop Trauma Surg.* (2022) 142:1753–62. 10.1007/s00402-021-03809-z 33570664

[68] Wang Y Ning N Li P Liu H. Analysis of the status and influencing factors of patient-reported outcomes after total hip arthroplasty. *J Chongqing Med Univ.* (2018) 43:1399–406.

[69] Çelik D Can C Aslan Y Ceylan HH Bilsel K Ozdincler AR. Translation, cross-cultural adaptation, and validation of the Turkish version of the Harris Hip Score. *Hip Int.* (2014) 24:473–9. 10.5301/hipint.5000146 25264204

[70] Christensen CP Althausen PL Mittleman MA Lee JA McCarthy JC. The nonarthritic hip score: reliable and validated. *Clin Orthop Relat Res.* (2003) 406:75–83. 10.1097/01.blo.0000043047.84315.4b 12579003

[71] Alshaygy I Alageel M Aljurayyan A Alaseem A Griffen A Arafah O et al. Cross-cultural adaptation and validation of the arabic version of the harris hip score. *Arthroplast Today.* (2022) 19:100990. 10.1016/j.artd.2022.07.006 36845291 PMC9947979

[72] Miley EN Casanova MP Cheatham SW Larkins L Pickering MA Baker RT. Confirmatory factor analysis of the hip disability and osteoarthritis outcome score (HOOS) and associated sub-scales. *Int J Sports Phys Ther.* (2023) 18:145–59. 10.26603/001c.67938 36793579 PMC9897000

[73] Murray DW Fitzpatrick R Rogers K Pandit H Beard DJ Carr AJ et al. The use of the Oxford hip and knee scores. *J Bone Joint Surg Br.* (2007) 89:1010–4. 10.1302/0301-620X.89B8.19424 17785736

[74] Machado RK Casagrande AA Pereira GR Vissoci JRN Pietrobon R Ferreira APB. Hip Disability and Osteoarthritis Outcome Score (HOOS): a cross-cultural validation of the Brazilian Portuguese version study. *Rev Bras Ortop.* (2019) 54:282–7. 10.1055/s-0039-1691764 31363282 PMC6597424

[75] Patel V Deshpande SV Jadawala VH Bhalsod D Sawant S. Evaluating short-term patient-reported outcome measures following total hip and knee replacement: a comprehensive review. *Cureus.* (2024) 16:e70468. 10.7759/cureus.70468 39479148 PMC11522172

[76] Ma Y Zhi X Zhang H. Investigation on the etiology of patients undergoing non-traumatic total hip arthroplasty in China. *J Orthop Surg.* (2022) 30:10225536221092114. 10.1177/10225536221092114 35400228

[77] Rizio AA Broderick LE White MK Quock TP. Content validation of the ATTR amyloidosis patient symptom survey: findings from patient and clinician cognitive debriefing interviews. *Patient Relat Outcome Meas.* (2020) 11:149–60. 10.2147/PROM.S264034 32904695 PMC7457571

