# Supplementary material for: Construction of a conceptual model and preliminary content development for patient-reported outcomes measurement after total hip arthroplasty: from a Chinese perspective
Source: Front Med (Lausanne). 2026 May 25;13:1753638. doi: 10.3389/fmed.2026.1753638 (PMC13243047; doi:10.3389/fmed.2026.1753638)
Supplement: Supplementary file 1 [file Data_Sheet_1.docx]

**Appendix 1 Basis for deletion of items with similar semantics and those that do not conform to the Chinese way of life**

| Dimension | Item | Basis for deletion |
| --- | --- | --- |
| Activities & functions | Outdoor gardening is not affected. | Does not conform to the Chinese way of life. |
|  | I can do treadmill training. |  |
|  | I can do lawn work or courtyard care work. |  |
|  | I can do exercises such as stretching and yoga. |  |
|  | I can participate in activities like bowling. |  |
|  | I can do some muscle strength training. |  |
|  | I can do weightlifting training. |  |
|  | I can get in and out of the bathtub easily. |  |
|  | I can participate in activities like golf. |  |
|  | I can participate in ball games such as billiards, badminton, and table tennis. |  |
|  | I need to walk at a very slow pace. | Repetition: There are too many items listed, such as walking, going up and down stairs, standing, lying down, and exercising. Many of these items have similar meanings. It is recommended to keep only one. |
|  | I can climb more than three floors of stairs without holding the handrail. |  |
|  | I can do housework such as sweeping the floor and mopping the floor by myself. |  |
|  | I can jump over some small obstacles. |  |
|  | I can stand with my feet together. |  |
|  | I can stand with one foot in front of the other. |  |
|  | I can sit on a high chair for more than half an hour. |  |
|  | I can take big steps when walking. |  |
|  | I can only lie in bed for most of the day and rarely get out of bed to move around. |  |
|  | I can make sharp turns when running fast. |  |
|  | I can do aerobic training. |  |
|  | Go shopping. |  |
| Pain | Have you ever felt pain due to your current illness? | "1. The pain dimension aims to evaluate the pain degree in different scenarios, and the results are presented in the form of ranked data. Items to be answered with "yes" or "no" are not applicable to this study; 2. There are too many items describing the pain degree when "sitting down", and there is semantic repetition; 3. The descriptions of pain type, intensity and frequency are too broad, and there is repetition with other items." |
|  | Was pain one of the symptoms when you were first diagnosed? |  |
|  | The pain has limited my activities. |  |
|  | I feel pain when I sit down. |  |
|  | The pain is more obvious when I sit on the ground. |  |
|  | The pain is not very obvious when I'm resting. |  |
|  | Type of pain |  |
|  | Pain intensity |  |
|  | Pain frequency |  |
| Quality of life | I can cook by myself. | "1. Doing housework has an inclusive relationship with items such as "cooking" and "washing clothes". It is sufficient to keep only one item; 2. There are too many items related to "medicine", "sleep", "rest", etc., and there is a problem of semantic repetition; 3. The items related to "using the toilet" have similar semantics." |
|  | I can wash my clothes by myself. |  |
|  | I can drive by myself. |  |
|  | I need to take a lot of different kinds of medicine. |  |
|  | My sleep has been really terrible in the past six months. |  |
|  | I need to take some sleeping pills to fall asleep. |  |
|  | I always wake up in the middle of the night when I sleep. |  |
|  | When doing something, I need to take a much longer rest. |  |
|  | I need to plan carefully before going out, and this bothers me a lot. |  |
|  | It takes me longer to use the toilet. |  |
| Perception& social support | I have a rich social life. | Semantic repetition |
|  | My friends and family are by my side when I need them. |  |
|  | My friends and family will support my decisions. |  |
| Mental | I'm afraid of injuring my hip joint again by participating in sports, and I feel frustrated about this. | Semantic repetition |
|  | I believe I perform excellently in sports. |  |
|  | My hip joint condition has deeply affected my sense of happiness. |  |
| Expectation | I hope to improve my ability to participate in social or recreational activities. | Semantic repetition |

**Appendix 2 The items that were deleted and considered to have a relatively low relevance**

| Dimension | Item |
| --- | --- |
| Satisfaction | During the medical treatment process, the medical staff made me feel relaxed and at ease. |
|  | During the medical treatment process, the medical staff were able to listen carefully. |
|  | During the medical treatment process, the medical staff showed sufficient concern. |
|  | During the medical treatment process, the medical staff fully understood my concerns. |
|  | During the medical treatment process, the medical staff were able to answer my questions thoroughly. |
|  | During the medical treatment process, the medical staff would discuss the rehabilitation strategies with me. |
|  | During the medical treatment process, different medical staff who treated and nursed me collaborated well and provided me with the best possible care. |
| Mental | In the past 4 weeks, I have felt depressed or sad. |
|  | In the past 4 weeks, I have felt calm and relaxed. |
|  | I have a feeling of fear, as if something terrible is about to happen. |
|  | I have a feeling of fear: |
|  | I feel restless, as if I have to keep moving: |
|  | I suddenly feel panicked: |
|  | I still enjoy the things I used to like in the past: |
|  | I can laugh and see the funny side of things: |
|  | I feel as if I have slowed down: |
|  | I have lost interest in my appearance: |
|  | I look forward to things with joy: |
|  | I can enjoy a good book, the radio or a TV program: |
|  | Sometimes my participation in local activities doesn't go well with my neighbors. |
|  | I can enjoy the pleasures that life brings. |
| Perception& social support | My friends and family understand me. |
|  | I can talk to my friends and family about my deepest problems. |
| Quality of life | I can feel my artificial joint in the following situations: |
|  | 1.When lying in bed at night? |
|  | 2.When I have been sitting on a chair for more than 1 hour? |
|  | 3.When I have been walking for more than 15 minutes? |
|  | 4.When I am taking a bath/shower? |
|  | 5.When I am traveling by vehicle? |
|  | 6.When I am climbing stairs? |
|  | 7.When I am walking on uneven ground? |
|  | 8.When I am getting up from a low seat? |
|  | 9.When I am standing for a long time? |
|  | 10.When I am doing housework or gardening? |
|  | 11.When I am walking/hiking? |
|  | 12.When I am doing my favorite sport? |
|  | I have a clear plan for my future. |
| Symptoms | I feel dizzy and nauseous. |
|  | I am troubled by constipation. |

**Appendix 3 Modification Status Table of Expert Consultation**

| Original Item | Modification suggestions for items | Revised Items |
| --- | --- | --- |
|  |  |  |
| F6. I can step over items on the floor, such as toys, sports equipment or small boxes. | Modify | I can step over the obstacles on the ground |
| F20. I can complete some heavy household chores (moving heavy boxes, scrubbing the floor, etc.). | Modify | I can complete some heavy household chores (such as carrying heavy objects, etc.). |
| F21. I can complete some light household activities (sweeping, vacuuming, cleaning, etc.). | Modify | I can complete easy household chores (such as sweeping the floor, mopping the floor, etc.) |
| F23. I can take care of the elderly or children (pushing a wheelchair, pushing a stroller, lifting). | Modify | I can take care of the elderly or children |
| F25. I can maintain a standing position. | Modify | I can stand still with both legs. |
| F30. When sitting on a low sofa, I need armrests or sit down heavily. | Modify | I can sit on a low sofa without holding onto anything |
| F35. I can get up from bed by myself. | Modify | I can get up from the bed by myself. |
| P2. I feel pain when walking on uneven roads. | Merge | P4 |
| P3. The pain is more obvious for me on hard roads. | Merge | P4 |
| Q6. I avoid talking about the joint problem. | Modify | I don't want to talk about issues related to the hip joint. |
| Q8. I always need others' help in daily life. | Modify | I need others' help in daily life. |
| Q13. I often feel worried. | Modify | I'm worried about problems with my hip joint. to MENTAL |
| PS11. I'm often afraid of becoming disabled. | Modify | I'm afraid of becoming disabled |
| M5. Because of the hip joint disease, I feel depressed and avoid going out. | Modify | Because of the hip joint disease, I'm in a bad mood and don't want to go out |
| M7. I feel nervous about participating in sports. | Modify | Because of the hip joint disease, I don't feel at ease mentally. |
| M10. I believe that I can participate in sports without any concerns. | Merge&Modify | M9,I believe that my hip joint won't have problems when participating in sports /working. |
| M16. Because of the hip joint disease, I'm anxious about my livelihood /daily life. | Modify | Because of the hip joint disease, I'm anxious about my daily life. |
| M18. Because of the hip joint disease, it's difficult for me to actively engage in various things. | Modify | Because of the hip joint disease, it's difficult for me to calm down and do things |
| M19 | Add | I'm worried about problems with my hip joint.（Mental ） |
| E4. I hope to no longer need to take medicine. | Merge | E1 |
| E9. I hope to improve my ability to go up and down stairs. | Modify | I hope to be able to go up and down stairs by myself. |
| E12. I hope to improve my ability to carry out activities at home (e.g., doing housework, gardening, etc.). | Modify | I hope to improve my ability to carry out activities at home (such as doing housework, working, etc.). |
| E14. I hope to improve my ability to carry out activities outside the home (e.g., shopping, etc.). | Modify | I hope to be able to go out for activities alone (such as shopping, walking, etc.). |
| E15. I hope to improve my ability to wear socks, stockings and shoes. | Modify | I hope to improve the ability of self-care in life (such as putting on shoes and socks by myself). |
| E17. I hope to improve my ability to carry out physical or sports activities. | Modify | I hope to improve the ability of physical activities. |
| E19. I hope to engage in occupational activities. | Modify | I hope to be able to return to work /participate in labor. |
| Sa1. Compared with before the surgery, the pain when I walk has been relieved a lot. | Merge | Sa2 |
| Sa7. Compared with before the surgery, I can sleep through the night now. | Merge | Sa8 |
| Sa17. After the surgery, my ability to carry out recreational activities has improved. | Merge | E18 |

**Appendix 4 Cognitive Debriefing Feedback and Item Revisions**

| **Items** | **Feedback Issues** | **Item Revisions** |
| --- | --- | --- |
| F6. I can step over items on the floor | What does "items" specifically refer to? | F6. I can step over items on the floor, such as toys, sports equipment, or small boxes. |
| F20. I can complete some heavy household chores | What does "heavy household"specifically refer to? | F20. I can complete some heavy household chores (moving heavy boxes, scrubbing the floor, etc.). |
| F21. I can complete some light household activities | What does "light household" specifically refer to? | F21. I can complete some light household activities (sweeping, vacuuming, cleaning, etc.). |
| F23. I can care for the elderly or children | What does "care" specifically refer to? | F23. I can care for the elderly or children (pushing a wheelchair, pushing a stroller, lifting). |
| M4.I sometimes get angry or feel nervous. | Is it associated with the disease? | M4. Because of the hip joint disease, I sometimes get angry or feel nervous. |
| M5.I feel depressed and avoid going out. | Is it associated with the disease? | M5. Because of the hip joint disease, I feel depressed and avoid going out. |
| M16.I'm anxious about my livelihood /daily life. | Is it associated with the disease? | M16. Because of the hip joint disease, I'm anxious about my livelihood /daily life. |
| M17.I'm dissatisfied with my health condition. | Is it associated with the disease? | M17. Because of the hip joint disease, I'm dissatisfied with my health condition. |
| M18.It's difficult for me to actively engage in various things. | Is it associated with the disease? | M18. Because of the hip joint disease, it's difficult for me to actively engage in various things. |
| Sa2.My pain has been relieved. | preoperative or postoperative? | Sa2. After the surgery, my pain has been relieved. |
| Sa4.I basically don't need to take painkillers anymore. | preoperative or postoperative? | Sa4. Compared with before the surgery, I basically don't need to take painkillers anymore. |
| Sa6.The pain at night has been relieved a lot. | preoperative or postoperative? | Sa6. Compared with before the surgery, the pain at night has been relieved a lot. |
| Sa9.The pain when I get into bed has been relieved a lot. | preoperative or postoperative? | Sa9. Compared with before the surgery, the pain when I get into bed has been relieved a lot. |
| Sa10.My muscle spasms are almost gone. | preoperative or postoperative? | Sa10. Compared with before the surgery, my muscle spasms are almost gone. |
| Sa12.My mood has improved a lot. | preoperative or postoperative? | Sa12. Compared with before the surgery, my mood has improved a lot. |
| Sa14.I feel generally very well. | preoperative or postoperative? | Sa14. Compared with before the surgery, I feel generally very well. |

**Supplementary Appendix1 The Search Strategy (Taking PubMed as an Example)**

((("Patient Outcome Assessment"[Mesh]) OR ((("patient" or "self") AND ("report" or "reported" or "reporting" or "rated" or "rating" or "based" or "assessed")) AND ("measure" or "measurement" or "scale" or "instrument" or "questionnaire"))) AND ((((((("structural validity" OR "factor analysis" OR "factor analyses") OR (("internal consistency") OR (cronbach AND (alpha OR alphas)))) OR ((cross-cultural or "cross cultural") AND (equivalence or validity))) OR ((test-retest OR (test AND retest)) AND (reliab*))) OR ((("measurement error") OR (((smallest OR minimal OR minimally OR clinical OR clinically) AND (important OR significant OR detectable)) AND (change OR difference))) OR ("limits of agreement"))) OR ("construct validity" OR concordance OR discriminative OR "known group")) OR (reproducib* or responsive*))) AND ((Hip) AND (arthroplasty or prosthesis or replacement or resurfacing))
